# Development of a Decellularized Urinary Bladder Matrix and Heparin‐Based Cryogel for Promoting Angiogenesis

**DOI:** 10.1002/mabi.202500028

**Published:** 2025-04-30

**Authors:** Dayeon Roo, Minkyu Lee, Sivashanmugam Amirthalingam, Kyung Min Ryu, Beom Seok Kim, Juan M. Melero‐Martin, Kyoung‐Ha So, Nathaniel S. Hwang

**Affiliations:** ^1^ Interdisciplinary Program in Bioengineering Seoul National University Seoul 08826 Republic of Korea; ^2^ Department of Chemical and Biological Engineering Seoul National University Seoul 08826 Republic of Korea; ^3^ Institute of Engineering Research Seoul National University Seoul 08826 Republic of Korea; ^4^ Bio‐MAX/N‐Bio Institute Institute of Bio‐Engineering Seoul National University Seoul 08826 Republic of Korea; ^5^ Department of Cardiac Surgery Boston Children's Hospital Boston MA 02115 USA; ^6^ Department of Surgery Harvard Medical School Boston MA 02115 USA; ^7^ Harvard Stem Cell Institute Cambridge MA 02138 USA

**Keywords:** cryogel, decellularization, neovascularization, urinary bladder matrix, vascular endothelial growth factor

## Abstract

Decellularized extracellular matrix(dECM)‐based scaffolds have demonstrated potential in promoting cellular migration and tissue regeneration. In this study, dECM‐based cryogel scaffolds are developed with sustained vascular endothelial growth factor (VEGF) release properties to enhance angiogenesis in ischemic tissues. VEGF plays a critical role in angiogenesis by stimulating cell proliferation and migration, but its therapeutic delivery remains challenging due to the need for precise dosing to avoid adverse effects. Cryogels, with their microporous structure, elasticity, and shape‐recovery characteristics, offer an ideal platform for controlled VEGF delivery. Using decellularized porcine urinary bladder matrix extracellular matrix (dECM) and heparin, a VEGF‐releasing cryogel scaffold is fabricated. The resulting dECM/heparin cryogel is a biocompatible scaffold capable of binding VEGF and releasing it over an extended period. This platform demonstrates significant angiogenic potential both in vitro and in a murine hindlimb ischemia model, highlighting its promise for therapeutic applications in tissue regeneration.

## Graphical abstract

A dECM‐based cryogel scaffold with sustained VEGF release is developed to promote angiogenesis in ischemic tissues. Fabricated from decellularized porcine urinary bladder matrix and heparin, the scaffold supports VEGF binding and prolonged release. The cryogel demonstrates enhanced angiogenic potential in vitro and in a murine hindlimb ischemia model, highlighting its potential for therapeutic tissue regeneration.

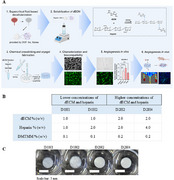

## 1 Introduction

Vascular diseases, such as peripheral vascular disease and critical limb ischemia, are the dysfunction in blood circulation and blood flow, resulting from the narrowing of blood vessels.^[^ ^1^ ^]^ This causes lack of blood supply to the peripheral tissues; thus hypoxia, inflammation, vascular damage, and muscle degeneration follow.^[^ ^1^ ^]^ Not only the conventional strategies including surgical and endovascular techniques, but also the recent advancements of gene therapy, cell therapy, and tissue engineering are being studied to enhance treatment of these complications. Vascular regeneration is a crucial process in tissue engineering as the vascular system supports the providence of nutrients and oxygen to the engineered tissue.^[^ ^2^ ^]^ Even though a few tissues are supplied with nutrients and oxygen through diffusion, engineered tissue with distant capillaries faces difficulties.^[^ ^2^ ^]^ Angiogenesis is the process of new blood vessel formation from a pre‐existing mature vascular bed composed of arteries, capillary networks, and veins.^[^ ^3^ ^]^ This involves a sequential and multistep process, which starts with an activation of a quiescent endothelium by angiogenic factors, such as vascular endothelial growth factor (VEGF), fibroblast growth factor (FGF), and angiopoietin‐2 (ANG‐2).^[^ ^4^ ^]^ Transforming growth factor‐β (TGF‐β), platelet‐derived growth factor B (PDGF‐B), ephrin‐B2, and NOTCH help maturation of a nascent blood vessel to be functional.^[^ ^4^ ^]^ Arteriogenesis causes vascular smooth muscle cells to enlarge and support the endothelial cell channels.^[^ ^4^ ^]^ In response to these vascular network formation processes, delivering the necessary growth factor and building vascular networks are needed in tissue engineering.

Among several ways to deliver the growth factor, scaffold‐based system allows incorporation of growth factors in the scaffold and local release at the specific site.^[^ ^5^ ^]^ Cryogel is a type of scaffold fabricated with chemical cross‐linking at subzero temperature. Due to its swelling property and highly interconnected porous structure, a cryogel shows the potential in biomedical applications: drug delivery, tissue engineering, and wound healing.^[^ ^6^ ^]^ Previous studies have focused on the use of heparin as a biomaterial moiety for growth factors to bind to.^[^ ^7^, ^8^, ^9^ ^]^ Heparin is a negatively charged glycosaminoglycan that enables binding of a growth factor through electrostatic interactions.^[^ ^10^ ^]^ Incorporating heparin into a cryogel resulted in a controlled release of a growth factor, such as VEGF, bone morphogenetic protein 4 (BMP4), and nerve growth factor (NGF).^[^ ^7^, ^8^, ^9^ ^]^ *Kim* et al. fabricated a cryogel by conjugating an amine group in gelatin type A and carboxyl group in heparin via 1‐ethyl‐3‐(3‐(dimethylamino)‐propyl) carbodiimide and sulfo‐hydroxysuccinimide (EDC/NHS).^[^ ^7^ ^]^ They could successfully make an injectable cryogel with rapid shape‐recovery property, which could be injected in vivo to enhance neovascularization.^[^ ^7^ ^]^ Due to its injectability, cryogel could be applied to the injury site non‐invasively. Additionally, *Park* et al. utilized chondroitin sulfate methacrylate (CS‐MA) and chitosan methacrylate (Chi‐MA) to form cryogel through radical polymerization initiated with ammonium persulfate (APS) and N,N,N’‐tetramethylethylenediamine (TEMED).^[^ ^11^ ^]^ They further added poly(ethylene glycol) diacrylate (PEGDA) as a supplementary polymer to stabilize the microporous structure. Pulverizing the developed cryogel and loading them with VEGF could enhance injection potential and angiogenesis.^[^ ^11^ ^]^ Not only loading of a single growth factor, but a sequential release of VEGF and BMP4 enabled bone regeneration.^[^ ^9^ ^]^ A double cryogel system consisted of VEGF‐loaded gelatin/heparin cryogel surrounding the BMP4‐loaded gelatin/chitosan cryogel in the core.^[^ ^9^ ^]^ As VEGF is initially released from gelatin/heparin cryogel, it resulted in angiogenesis in an in vivo cranial defect model.^[^ ^9^ ^]^ A subsequent sustained release of BMP4 from gelatin/chitosan cryogel further facilitated bone regeneration.^[^ ^9^ ^]^ Even though the biomaterials that had been previously used are biocompatible, biodegradable, and bioactive, providing the host cells with a growth factor in extracellular matrix would further enhance the function of the biomaterial. Recent advancements in biomaterials include decellularization techniques. Decellularization refers to the removal of cellular components from the tissues, leaving an acellular extracellular matrix (ECM). Among different types of tissues, porcine urinary bladder matrix (UBM) contains the structural elements such as collagen, glycosaminoglycans, and growth factors that can improve cell growth, proliferation, and attachment.^[^ ^12^ ^]^ In addition to the structural elements, another advantage of UBM is the minimal immunogenicity.^[^ ^12^ ^]^ Urinary bladder wall is divided into four layers, which are the urothelium, the lamina propria, the muscular layer, and the serosal layer.^[^ ^13^ ^]^ Lamina propria is highly porous and rough in texture that is appropriate for cell infiltration, and the intact epithelial basement membrane composes of proteins such as collagen IV and VII that allow cellular anchoring and migration. By preserving these components from the basement membrane with a supercritical carbon dioxide decellularization technique, we expected this biomaterial to be biocompatible and nonimmunogenic.^[^ ^14^ ^]^

Here, we have utilized the supercritical fluid‐based decellularized porcine urinary bladder matrix extracellular matrix (dECM) and heparin to fabricate a cryogel (dECM/heparin). This cryogel is capable of anchoring VEGF, thereby providing a sustained release and resulting in angiogenesis both in vitro and in vivo. First, we characterized the cryogels with different concentrations of dECM and heparin. Each condition was assessed for cytotoxicity and cellular proliferation. We then examined the effect of angiogenesis in vitro via cell scratch assay, tube formation assay, and RT‐qPCR. Based on the in vitro result, we have tested its effect in vivo in a mouse hindlimb ischemic model to examine the blood perfusion change.

## 2 Results

### 2.1 Fabrication of dECM/Heparin Cryogel

**Figure 1:**
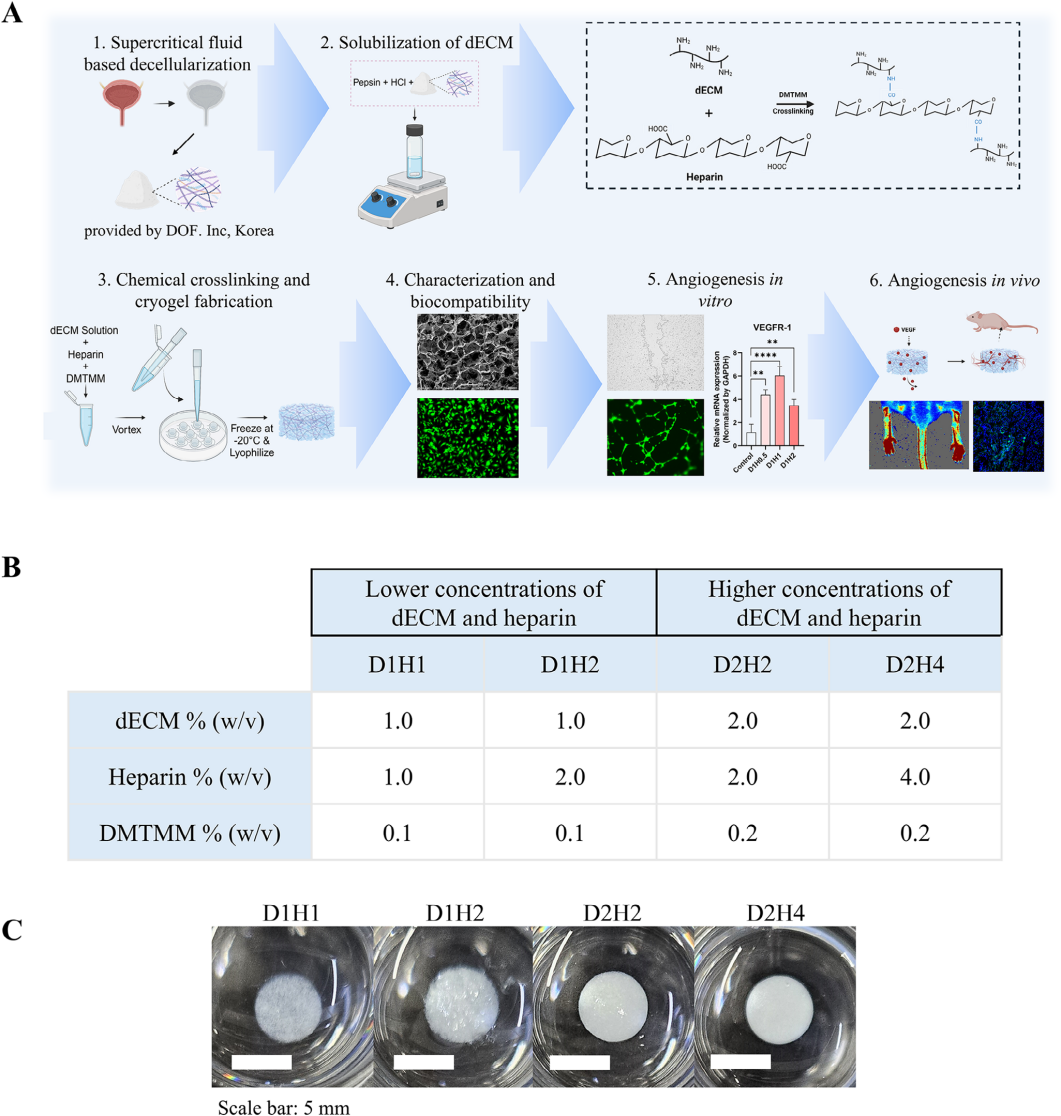
Fabrication of dECM/heparin cryogel. A) Overall schematic illustration of the study. B) Composition of dECM and heparin in each cryogel condition. C) Macroscopic images of dECM/heparin cryogels. The scale bar was 5 mm.

We utilized decellularized porcine urinary bladder matrix extracellular matrix, which was decellularized with a supercritical fluid‐based method. Cross‐linking with heparin, dECM/heparin cryogel was fabricated at −20 °C for 24 h and lyophilized overnight to have a porous network. In order to examine different types of cryogels using different concentrations of dECM and heparin, we fabricated cryogels with lower dECM and heparin concentrations and higher dECM and heparin concentrations. Cryogels with lower dECM and heparin concentrations were composed of 1% (w/v) dECM (D1) and 1:1 and 2:1 w/w ratio of heparin (H1 and H2). 1:10 w/w ratio of DMTMM to dECM was used as a cross‐linker. As a result, cryogels with lower dECM and heparin concentrations were named D1H1 and D1H2. Cryogels with higher dECM and heparin concentrations were composed of 2% (w/v) dECM (D2) and 1:1 and 2:1 w/w ratio of heparin (H2 and H4). Similarly, a 1:10 w/w ratio of DMTMM to dECM was used, and the final products were D2H2 and D2H4. Detailed composition is presented in **Figure** **1**B. When swollen, cryogels with lower dECM and heparin concentrations were more transparent in color than cryogels with higher dECM and heparin concentrations; however, they all could remain their shapes (Figure 1C).

### 2.2 Characterization of dECM/Heparin Cryogel

**Figure 2:**
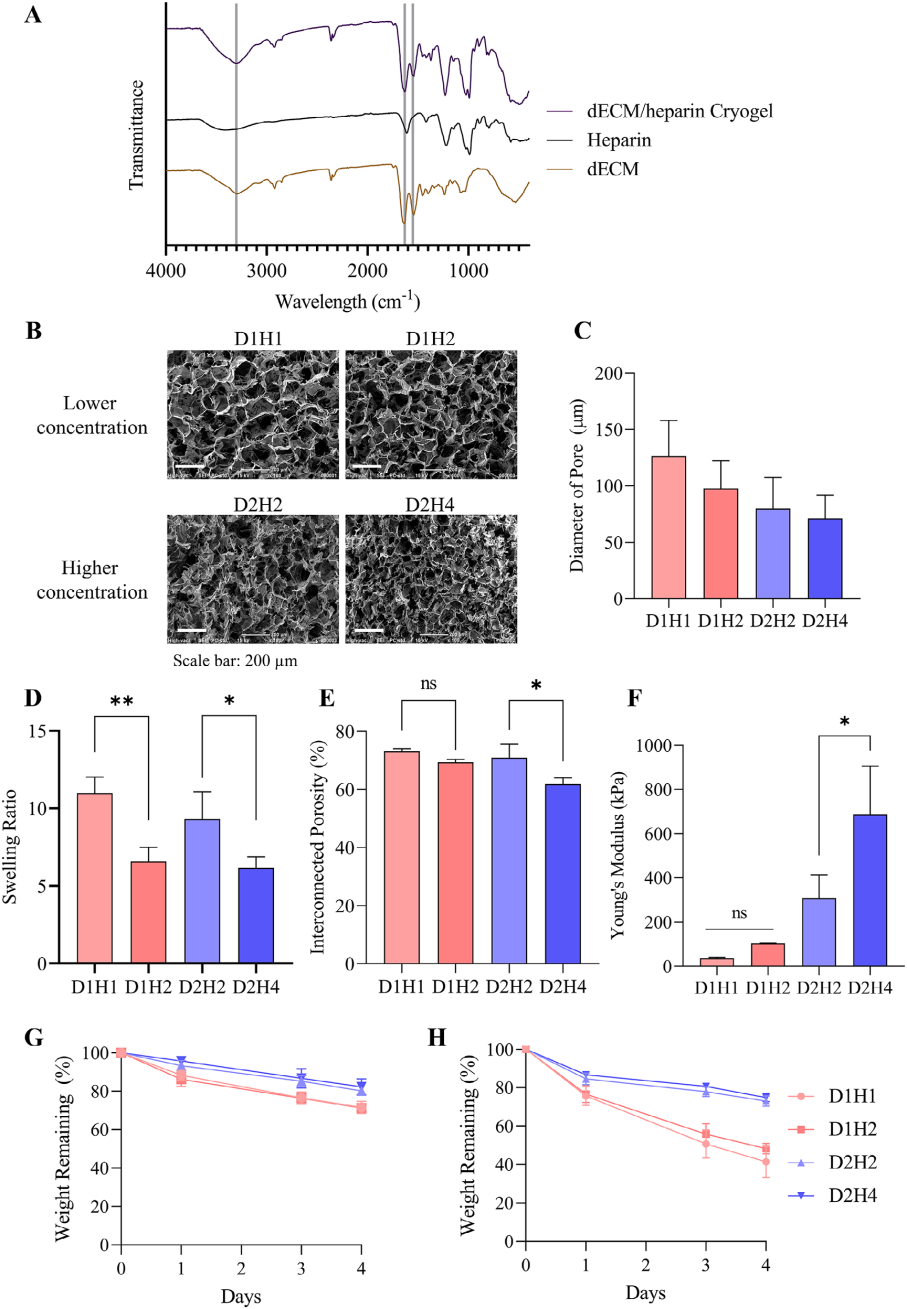
Characterization of dECM/heparin cryogel. A) FT‐IR transmittance analysis of dECM/heparin cross‐linking network. B) Mini‐SEM images showing microstructure of porous cryogels and C) the quantification of dECM/heparin cryogel analyzed by ImageJ. D) Swelling ratio, E) interconnected porosity, and F) Young's modulus of cryogels. Degradation rate at G) 0.2 unit mL^−1^ of collagenase and H) 1 unit mL^−1^ of collagenase. The scale bar was 200 µm. * indicated *p* < 0.05 and ** indicated *p* < 0.01.

Cross‐linking of dECM and heparin was analyzed with FT‐IR. dECM alone, heparin alone, and dECM/heparin cryogel were analyzed, and the transmittance peaks were evaluated (**Figure** **2**A). Transmittance peaks at 3300 cm^−1^ showed a stretching frequency of N─H in amide group, and 1630 cm^−1^ showed a C═O stretching of an amide I. Lastly, the peak at 1550 cm^−1^ showed N─H bending of an amide II. Cryogels are characterized to have a porous structure with an interconnected network. In order to examine the pore structure of cryogels, we have looked into the mini‐SEM images of a cross‐sectioned cryogel (Figure 2B).

As the concentration of heparin increases in both cryogel groups, the pore sizes were more homogeneous (Figure S1, Supporting Information). Cryogels with lower dECM and heparin concentrations had bigger pore sizes than cryogels with higher dECM and heparin concentrations (Figure 2C). In addition to a structural component, we figured out the mechanical properties, such as swelling ratio, interconnected porosity, and Young's modulus, of the scaffolds. Both groups of cryogels showed a decrease in swelling ratio and interconnected porosity as the concentration of heparin increased (Figure 2D,E). This is related to the pore size of the scaffold. As the pore size decreases with the increasing heparin concentration, scaffolds are less likely to swell and contain an interconnected porosity. Not only does the concentration of heparin contribute, but those properties are also related to the extent of cross‐linking. Cryogels with higher dECM and heparin concentrations showed a lower swelling ratio and interconnected porosity when compared to cryogels with lower dECM and heparin concentrations. However, their Young's modulus were significantly higher than that of the cryogels with lower dECM and heparin concentrations (Figure 2F). Increasing the concentration of heparin also affected the increase of Young's modulus (D1H1: 35.74 ± 4.92 kPa, D1H2: 103.74 ± 0.83 kPa, D2H2: 308.97 ± 104.93 kPa, D2H4: 686.76 ± 218.65 kPa). Moreover, the degradation rate of the cryogels were measured in 0.2 and 1 unit mL^−1^ of collagenase (Figure 2G,H). Cryogels fabricated with lower concentrations of dECM and heparin (D1H1 and D1H2) degraded faster than cryogels with higher concentrations of dECM and heparin (D2H2 and D2H4). Furthermore, when we used higher concentration of collagenase (1 unit mL^−1^), the degradation rate was faster compared to 0.2 unit mL^−1^.

**Figure 3:**
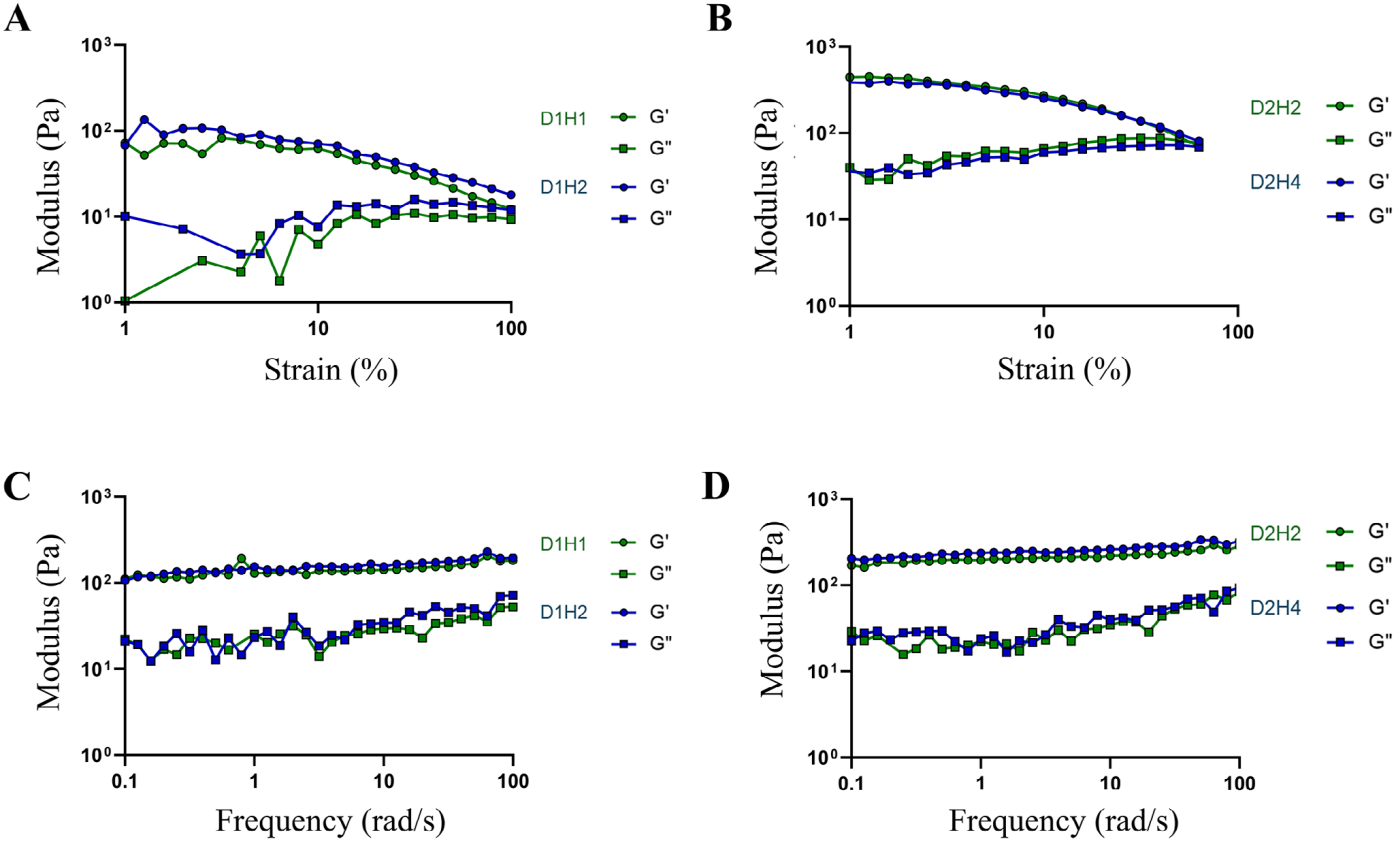
Rheological properties of dECM/heparin cryogel. Storage modulus (G’) and loss modulus (G″) of dECM/heparin cryogels on A,B) strain sweep measured at 1 Hz of frequency and C,D) frequency sweep measured at 5% of strain.

In addition to the mechanical properties, rheological properties such as strain‐sweep and frequency‐sweep were assessed by measuring the rheological moduli: storage modulus (G’) and loss modulus (G″). Rheological properties display the deformation and cross‐linking degree of the material. First, we carried out the strain‐sweep test, which measures the stability of network against deformation with the increasing deflection at the constant frequency. For all the cryogels, G’ value was higher than G″, indicating that the cryogels had a gel‐like or solid structure and not liquid (**Figure** **3**A,B). G’ and G″ values increased with the increasing heparin concentration in a cryogels with lower dECM and heparin concentrations (Figure 3A). However, it was difficult to observe this trend in cryogels with higher dECM and heparin concentrations (Figure 3B). G’ and G″ values of cryogels with higher dECM and heparin concentrations were greater than that of lower concentration cryogels, which represented a stiffer property. Second, we conducted the frequency‐sweep test with a constant strain of 5%. Frequency‐sweep test observes the cross‐linking degree and inner structure of a cryogel by measuring G’ and G″ in the range of non‐deformative strain range. Here also shows a G’ > G″, and the values are slightly increasing in both cryogel groups (Figure 3C,D). G’ and G″ of cryogels with higher dECM and heparin concentrations displayed enhanced moduli than lower concentration cryogels. This indicated the cross‐linking degree of cryogels with higher dECM and heparin concentrations was higher than that of lower concentration cryogels.

With characteristics, cryogels are known to recover rapidly and have an injectability property. We confirmed this by squeezing the cryogel with forceps. All cryogels could successfully recover their shapes after being squeezed (Figure S2A, Supporting Information). For the injectability test, we used a 17G needle syringe to inject cryogel (Figure S2B, Supporting Information). Even though all cryogels could be injected and recovered their shapes, cryogels with lower dECM and heparin concentrations showed a better performance in shape recovery as higher concentration cryogels were slightly dismantled and had dECM debris after injection (Figure S2C,D, Supporting Information). The weight loss after injection was greater for higher concentration cryogels as they were harder to be injected (Figure S2E, Supporting Information). This suggested that lower concentration cryogels were more suitable for injection.

### 2.3 Biocompatibility of dECM/Heparin Cryogel

**Figure 4:**
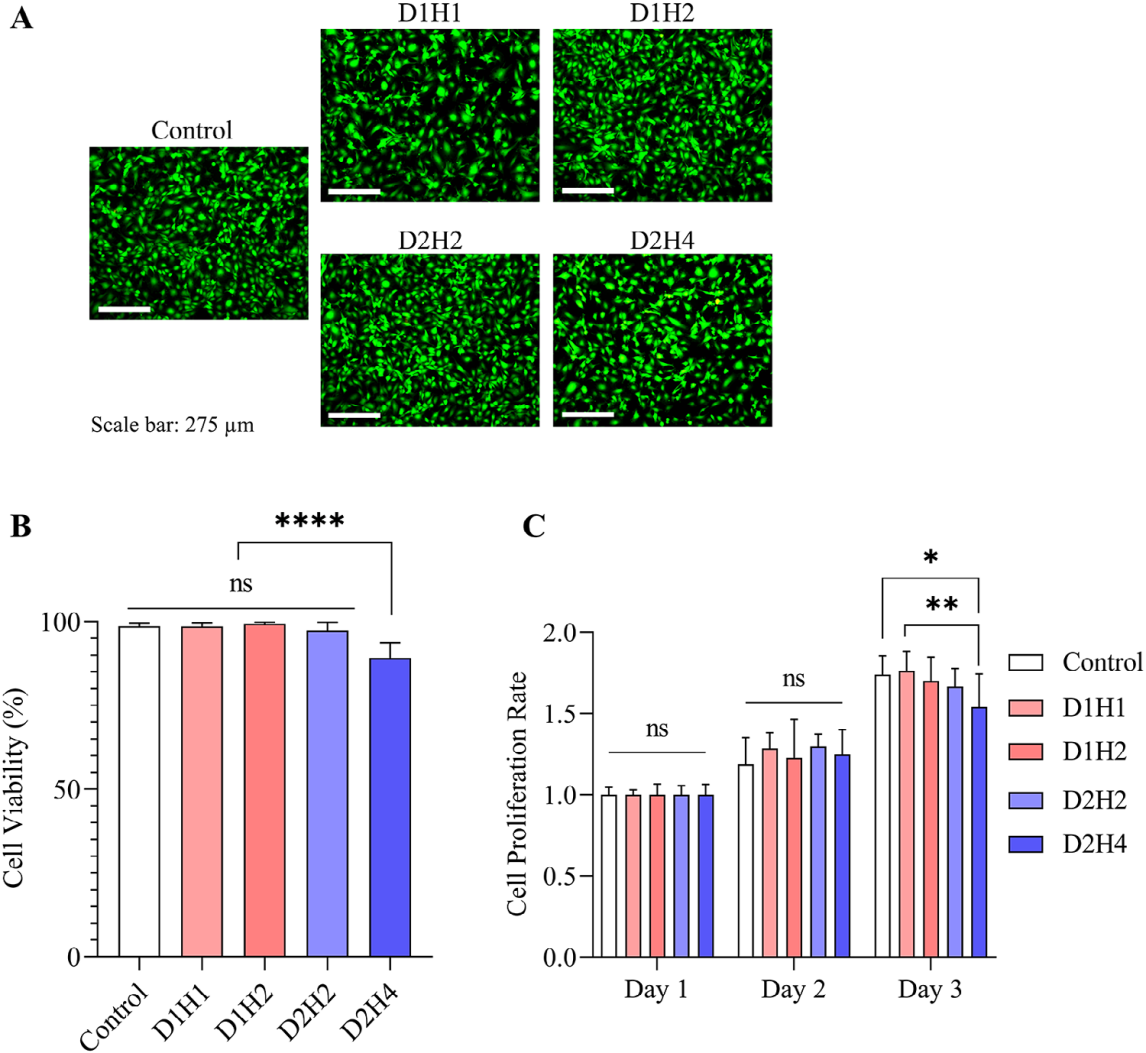
Biocompatibility of dECM/heparin cryogel. A) Cytotoxicity test with LIVE/DEAD assay where live cells were stained in green and dead cells were stained in red. B) Quantification of cell viability. The scale bar was 275 µm. C) Cellular proliferation rate measured with cell counting kit‐8 assay. * indicated *p* < 0.05, ** indicated *p* < 0.01, and **** indicated *p* < 0.0001.

Cytotoxicity and cell proliferation ability with the dECM/heparin cryogels were measured with LIVE/DEAD assay and Cell Counting Kit‐8 assay. LIVE/DEAD solution stained the live cells with calcein‐AM in green and dead cells with ethidium homodimer‐1 in red (**Figure** **4**A). Conditioned medium that contained cryogels was provided to HUVECs, and viability was measured after 24 h. Both groups of cryogels were not cytotoxic to cells, but D2H4 cryogel showed a lower viability compared to other groups (Control: 98.66 ± 0.93%, D1H1: 98.57 ± 1.10%, D1H2: 99.41 ± 0.43%, D2H2: 97.38 ± 2.53%, D2H4: 89.18 ± 4.56%) (Figure 4B). There was no statistical significance among the studied groups, except for the D2H4, which possessed lower cell viability compared to other groups.

For cell proliferation, we used the same conditioned medium and cultured HUVECs for three days. We have calculated the number of cells in each condition every day and examined the cell proliferation rate. Until day 2, cells with dECM/heparin cryogels proliferated more than the control group, but cryogels with higher dECM and heparin concentrations proliferated in a slower rate in day 3 than control and lower concentration cryogels (Figure 4C).

### 2.4 In Vitro Angiogenesis Effects of dECM/Heparin Cryogel

**Figure 5:**
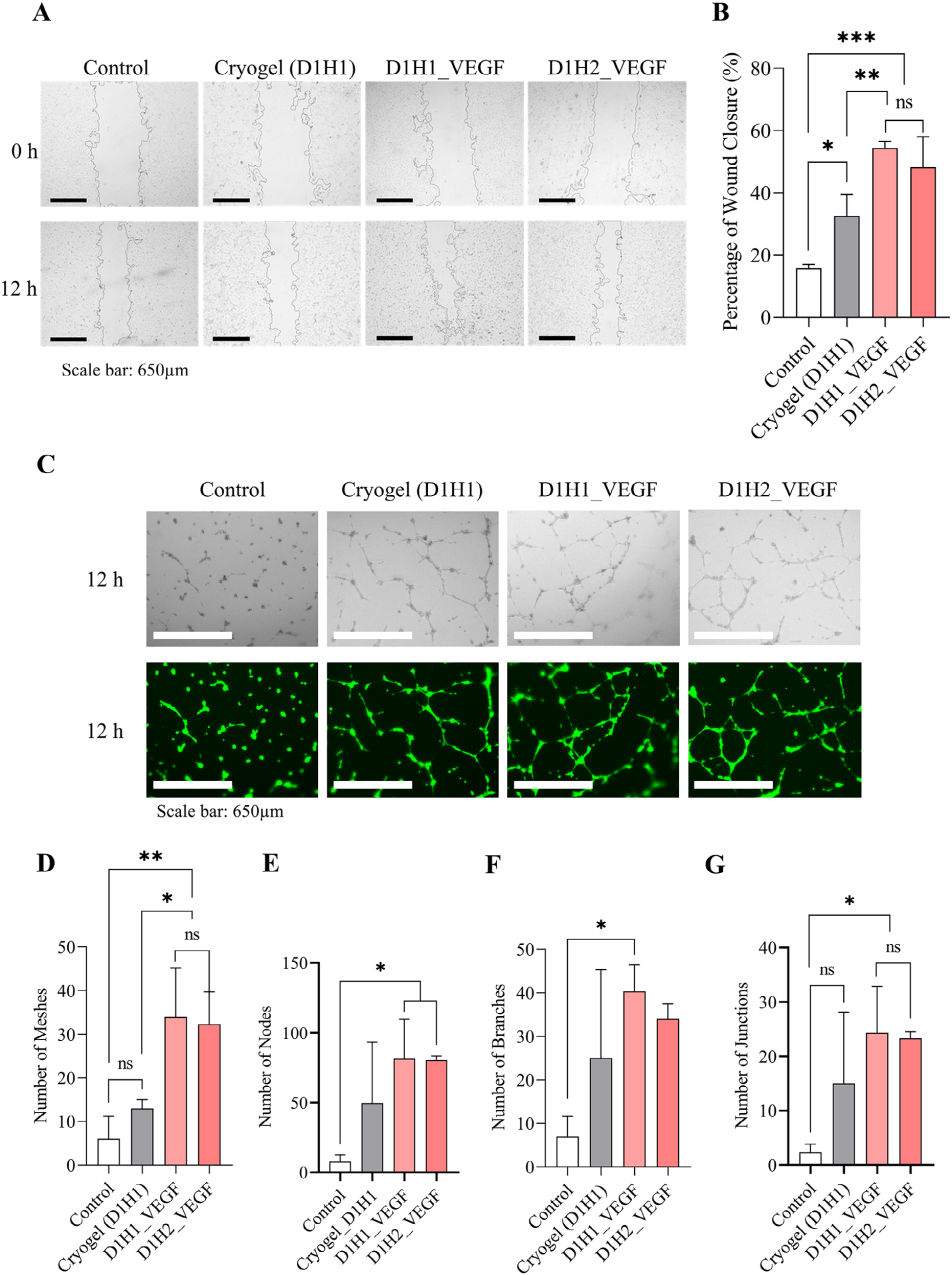
Angiogenic potential in vitro on HUVECs in response to dECM/heparin cryogel loaded with VEGF. A) Representative bright field images of cell scratch assay at 0 and 12 h. B) Quantification of the wound closure analyzed by ImageJ. C) Representative images of tube formation assay stained with calcein‐AM after 12 h. D–G) Quantification of the tube formation assay including D) number of meshes, E) number of nodes, F) number of branches, and G) number of junctions. The scale bar was 650 µm. * indicated *p* < 0.05, ** indicated *p* < 0.01, and *** indicated *p* < 0.001.

Angiogenic potential of VEGF‐loaded dECM/heparin cryogel were assessed with cell migration assay, tube formation assay, and RT‐qPCR. VEGF165, the most abundant and functional isoform of VEGF‐A monomers, plays an important role in regulation of the endothelial cell function.^[^ ^16^ ^]^ As the guidance cue, such as VEGF, is present in dECM/heparin cryogel, we expected this would cause cell migration and tube formation, which are commonly used to evaluate the angiogenic potential in vitro. We compared the VEGF‐unloaded and VEGF‐loaded dECM/heparin cryogel groups with negative control after giving the conditioned media. To avoid confusion, VEGF‐loaded dECM/heparin cryogel is labeled with _VEGF. The conditioned media were made by filtering EBM with 2% FBS and 1% anti‐anti after incubating with cryogel or VEGF‐loaded cryogel (D1H1_VEGF or D1H2_VEGF) for 24 h. We expected the conditioned medium to contain VEGF and dECM contents released from the cryogel. **Figure** **5**A depicts that the percentage of wound closure was 54.41 ± 2.08% for D1H1_VEGF and 48.28 ± 9.71% for D1H2_VEGF, which were higher than a VEGF‐unloaded dECM/heparin cryogel (32.57 ± 6.82%). The presence of VEGF in the cryogel enhanced endothelial cell migration. When compared to negative control (13.30 ± 9.04%), a cryogel without VEGF also showed a higher percentage of wound closure. However, the difference between the control group and cryogel was not significant nor did the difference between D1H1_VEGF and D1H2_VEGF (Figure 5B).

**Figure 6:**
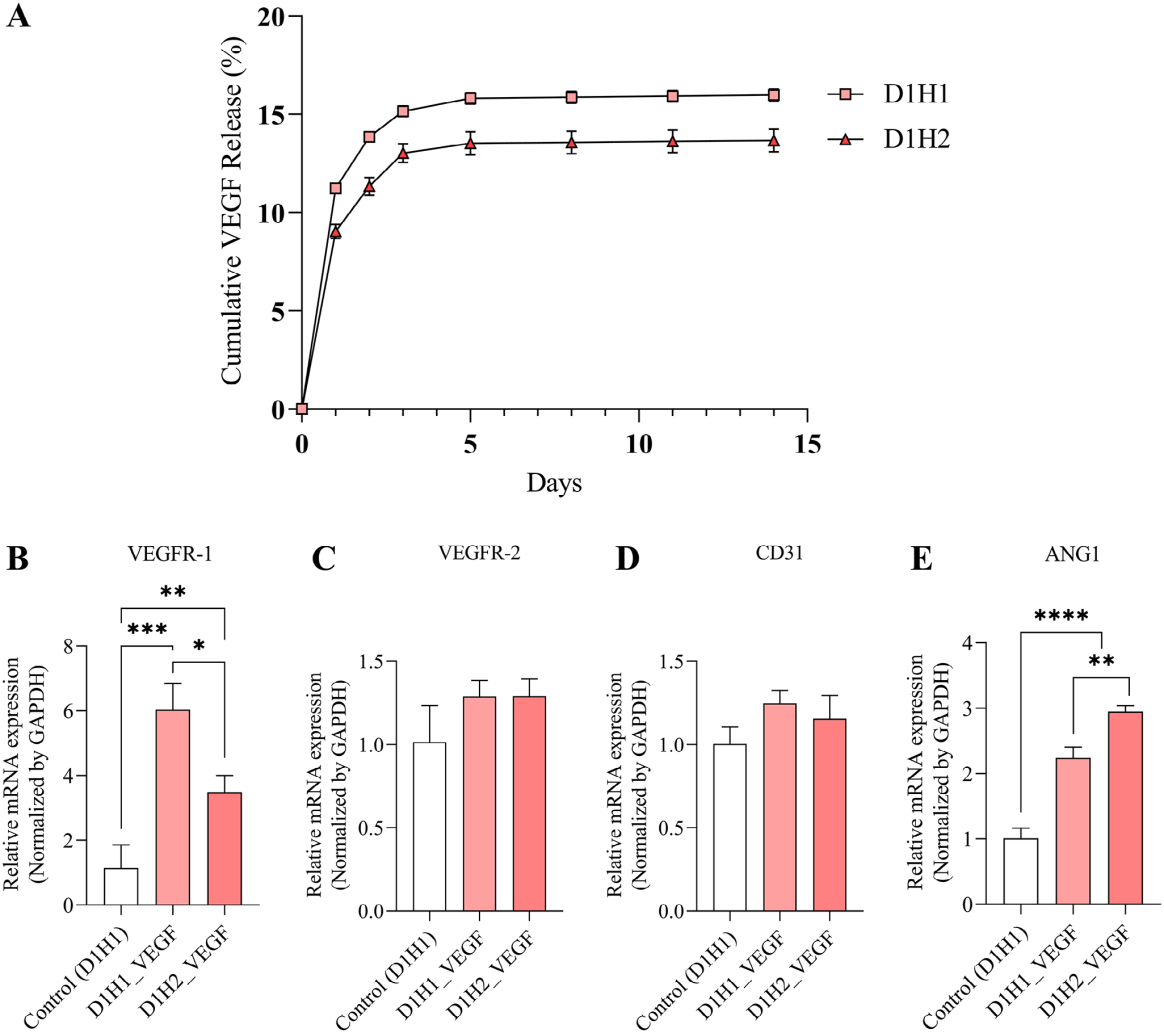
Angiogenic potential in a conditioned medium collected from dECM/heparin cryogel loaded with VEGF. A) Cumulative VEGF release rate from dECM/heparin cryogels measured by VEGF ELISA kit. B–E) Analysis on angiogenesis‐related gene expressions of HUVECs with dECM/heparin cryogels loaded with VEGF by RT‐qPCR. * indicated *p* < 0.05, ** indicated *p* < 0.01, *** indicated *p* < 0.001, and **** indicated *p* < 0.0001.

In addition to cell scratch wound healing assay, a tube formation assay was done to quantitatively analyze pro‐angiogenic potential in vitro. Endothelial cells, upon exposure to angiogenic signal, such as VEGF, align to form lumen‐containing tubules.^[^ ^17^ ^]^ In Figure 5C, VEGF‐loaded groups (D1H1_VEGF and D1H2_VEGF) showed a tube formation after 12 h of incubation in Matrigel. Similar to cell migration, VEGF‐loaded dECM/heparin groups (D1H1_VEGF and D1H2_VEGF) showed more meshes, nodes, branches, and junctions than VEGF‐unloaded dECM/heparin groups (Figure 5D–G). Among VEGF‐loaded cryogels, tube formation occurred slightly less for the cryogel with a higher concentration of heparin (D1H2_VEGF) although there was no significant decrease. This trend of having higher pro‐angiogenic potential in a lower heparin concentration may be explained by the amount of VEGF released. While both D1H1_VEGF and D1H2_VEGF demonstrated a controlled release of VEGF over five days, D1H1_VEGF released more VEGF than D1H2_VEGF. D1H1_VEGF released 15.81 ± 0.30% of the initially loaded VEGF, and D1H2_VEGF released 13.52 ± 1.08% (**Figure** **6**A), suggesting that the higher VEGF release from D1H1_VEGF contributed to its greater angiogenic activity. We postulate that heparin helps anchor VEGF to the scaffold, but heparin with a higher concentration limits the release of bound VEGF.

Furthermore, we examine the gene expression changes of endothelial cells upon exposure to VEGF‐unloaded and VEGF‐loaded dECM/heparin cryogels. Gene expressions of vascular endothelial growth factor receptor 1 (*VEGFR‐1; FLT1*), vascular endothelial growth factor receptor 2 *(VEGFR‐2; KDR)*, platelet endothelial cell adhesion molecule (*PECAM‐1; CD31*), and Angiopoietin‐1 (*ANG1*) were tested after incubating endothelial cells with the cryogel for three days. VEGFR‐1 is expressed in endothelial cells and contribute to a regulatory function in VEGF signaling.^[^ ^18^ ^]^ ANG1 is known as an angiogenesis regulator at the late stage and contributes to vessel stability and maturation.^[^ ^19^ ^]^ CD31 is found on the surface of endothelial cells and involved in the endothelial cell–cell adhesion for neovascularization.^[^ ^20^ ^]^ The relationship between heparin concentration and these angiogenic gene expressions could not be drawn from the results, but VEGF‐loaded cryogels (D1H1_VEGF and D1H2_VEGF) showed an upregulation of those four genes compared to VEGF‐unloaded cryogel (control) (Figure 6B–E). Considering the characteristics and in vitro angiogenic potential of the lowly crosslinked cryogels, we selected D1H1 as an optimal cryogel composition among others. D1H1 had a homogenous pore structure with a large pore size and adequate stiffness that could result in high swelling ratio and interconnected porosity. Additionally, it can hold VEGF so that it does not burst release in a short period of time and can stimulate cell migration and tube formation. For these reasons, we conducted further in vivo experiments with D1H1 cryogel.

### 2.5 Neovascularization Potential of dECM/Heparin Cryogel In Vivo

**Figure 7:**
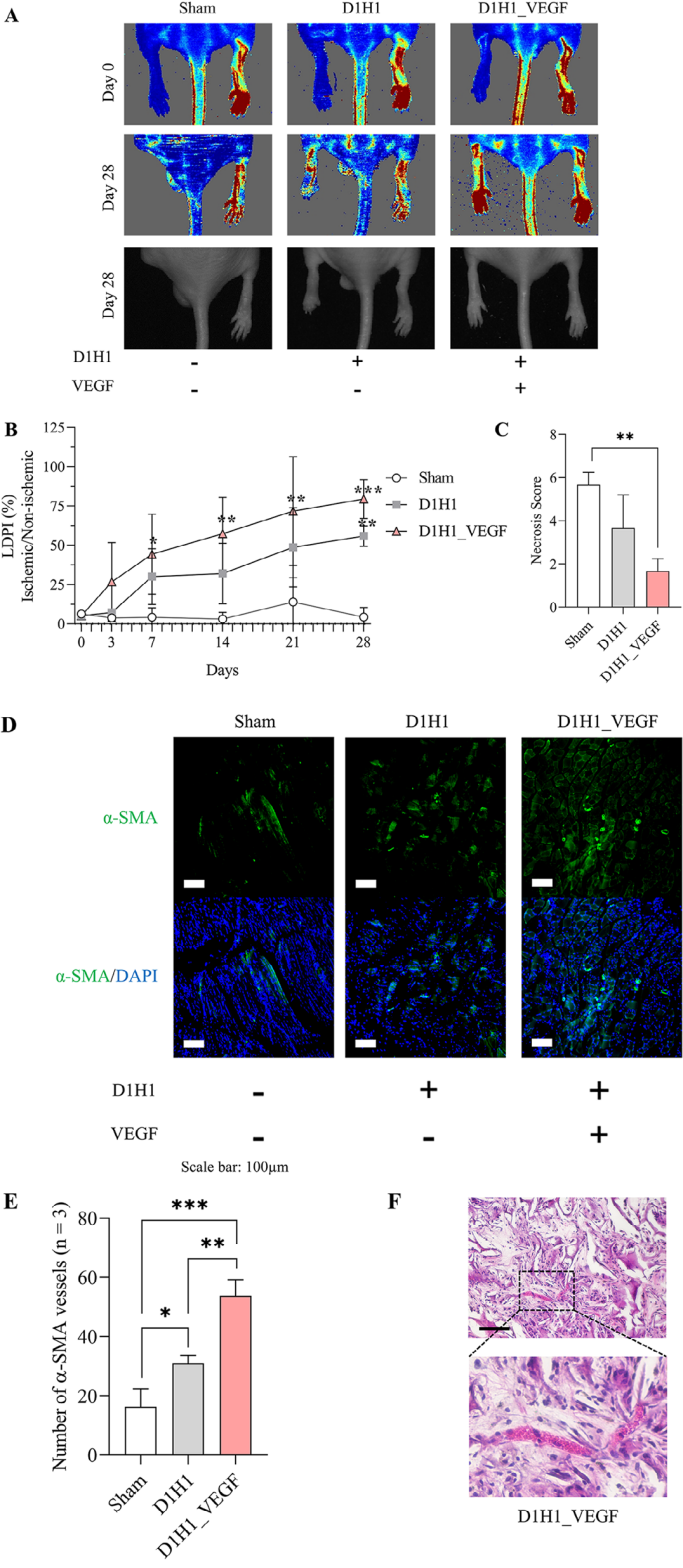
Angiogenic potential in vivo in an ischemic model. A) Laser Doppler blood perfusion images of ischemic (left) and non‐ischemic (right) hindlimbs on day 0 post‐surgery and on day 28. B) Quantification of blood perfusion by laser Doppler perfusion imaging (LDPI) ratio. C) Necrosis score indicating the salvage score of the hindlimb on day 28. The score ranges from full recovery (score 1) to limb amputation (score 6). D) Immunohistochemistry with α‐smooth muscle cells (α‐SMA) to show blood vessels. E) Quantification of blood vessels. F) Ex vivo H&E staining of a dECM/heparin scaffold with VEGF on day 28. The scale bar was 100 µm. * indicated *p* < 0.05, ** indicated *p* < 0.01, and *** indicated *p* < 0.001.

The neovascularization potential of a VEGF‐loaded dECM/heparin cryogel (D1H1_VEGF) was studied in a mouse hindlimb ischemia model. The mouse hindlimb ischemia model was induced by ligating a femoral artery. To evaluate the changes in blood perfusion in an ischemic limb, we have treated an ischemic limb with D1H1 and D1H1_VEGF. Blood perfusion after the surgery explicitly showed that all groups were induced with hindlimb ischemia as the blue color on the limb represented a blockage of the blood flow (**Figure** **7**A). Blood perfusion was tracked on days 3, 7, 14, 21, and 28, and the corresponding laser Doppler perfusion index (LDPI) was plotted (Figure 7B). Until day 7, there was a higher blood perfusion in D1H1 and D1H1_VEGF groups when compared to sham group. From day 14 to 28, D1H1 and D1H1_VEGF groups showed a significantly higher blood perfusion than sham group. Especially for D1H1_VEGF, the blood perfusion of the ischemic limb was 79.5% recovered. The salvage score on day 28 also supported this result (Figure 7C). Not only did D1H1_VEGF cryogel show a limb recovery, but D1H1 cryogel itself helped in the limb salvage to a certain extent.

After 28 days, mice were sacrificed, and the tissue at the injury site was collected for immunostaining of α‐SMA that stains the smooth muscle cells in the blood vessels. Figure 7D represents the α‐SMA staining with green fluorescence and DAPI staining with blue fluorescence of the cross‐section of the hindlimb tissue. With respect to the previous results, D1H1_VEGF group showed the highest number of vessels, and D1H1 group followed. Even though D1H1 group exhibited a low degree of angiogenesis, it showed a significant number of vessels when compared to sham group, which corresponds to the result in Figure 7B (Figure 7E). This further supports the regeneration and angiogenesis potential of UBM dECM.

We investigated cell infiltration in the VEGF‐loaded dECM/heparin cryogels with H&E staining. Hematoxylin‐stained cell nucleus in purple, and eosin‐stained extracellular matrix and cytoplasm in pink. VEGF‐loaded cryogel integrated in the host tissue, and there showed cell infiltration into a scaffold after 28 days as cell nuclei stained in purple are bound to a surface of the scaffold (Figure 7F). Further analysis is needed to distinguish which kinds of cells were infiltrated into a scaffold.

## 3 Discussion

Scaffold‐based tissue engineering utilizes a scaffold with either cells or biomolecules or both to facilitate the regeneration of tissues. In this study, we loaded VEGF in a cryogel scaffold constructed with dECM and heparin to regenerate the vascular system. The amine group of dECM and the carboxyl group of heparin were cross‐linked via 4‐(4,6‐dimethoxy‐1,3,5‐triazin‐2‐yl)‐4‐methylmorpholinium chloride (DMTMM). As dECM primarily consists of proteins that possess free amine groups, and heparin contains carboxyl group, we employed DMTMM, a cross‐linker capable of reacting with amine and carboxyl groups to form amide linkage, hence utilized to fabricate the cryogel.^[^ ^21^, ^22^, ^23^ ^]^ Unlike 1‐ethyl‐3‐(3‐dimethylaminopropyl)carbodiimide (EDC) and sulfo‐hydroxysuccinimide (NHS), DMTMM does not need a pH control during the chemical reaction and easy to use.^[^ ^23^ ^]^ With the ease of fabrication, we further characterized the scaffolds with different concentrations of dECM and heparin.

The pore size of a scaffold is crucial in tissue engineering as it can vary the extents of cell migration, attachment, and growth.^[^ ^24^ ^]^ Small pores hinder cell infiltration into the center of a scaffold as there is less diffusion of nutrients and oxygen.^[^ ^24^ ^]^ Large pores hinder cell attachment as there is limited surface area.^[^ ^24^ ^]^ Previous studies have suggested that scaffolds with large sized pores (160–270 µm) are superior in rapid and extensive angiogenesis.^[^ ^25^ ^]^ Considering this, we hypothesized that cryogels fabricated with a low concentration of dECM and heparin that consisted of a large pore size would be suitable for angiogenesis study. We observed that D1H1 and D1H2 cryogels had a bigger pore size with a higher swelling ratio and interconnected porosity and lower Young's modulus than dECM/heparin cryogels fabricated with higher concentration of dECM and heparin (D2H2 and D2H4). An increase in concentration of heparin also reduced the pore size, swelling ratio, and interconnected porosity while increasing Young's modulus. Even though increasing stiffness is related to an increase in cell migration and sprouting, the cells do not tend to form a tube when the stiffness of a scaffold is too high or too low.^[^ ^26^ ^]^ Soft tissues such as neutral tissues have 1–4 kPa, and the tissues that are exposed to high mechanical loading exhibit greater stiffness; for example, the heart is 10–15 kPa.^[^ ^27^ ^]^ Since angiogenesis is an essential function that the scaffold needs in vivo, D1H1 and D1H2 cryogels were favored.

Before loading any growth factor, we examined the biocompatibility of dECM/heparin cryogel. As described in 3.3., all cryogels were biocompatible while D2H4 showed a lower viability and cell proliferation. It was previously studied that a high concentration of heparin inhibits cell proliferation and growth, and a higher concentration may result in cell apoptosis.^[^ ^28^ ^]^ In addition, a negative charge of heparin may hinder cell migration and adhesion (Figure S3, Supporting Information).^[^ ^8^ ^]^ Therefore, we selected D1H1 and D1H2 for further in vitro angiogenesis study. When we loaded VEGF for further angiogenesis study, morphology of a cryogel changed to a small extent. VEGF‐loaded cryogel (D1H1_VEGF) was smaller than VEGF‐unloaded cryogel (D1H1) (Figure S4A, Supporting Information). Furthermore, VEGF loading resulted in lower swelling ratio and interconnected porosity. We hypothesize that these results were influenced by lyophilization of D1H1_VEGF and VEGF‐heparin binding. VEGF‐bound dECM/heparin cryogels showed higher angiogenic effects in HUVECs than VEGF‐unbound dECM/heparin cryogels when cell scratch wound healing assay and tube formation assay were conducted. Introduction of VEGF in endothelial cell is known to stimulate cell proliferation, migration, and angiogenesis.^[^ ^29^ ^]^ Similarly, our RT‐qPCR results pointed out higher gene expressions of VEGFR‐1, VEGFR‐2, PECAM‐1, and ANG1. This corresponded to previous studies with VEGF.^[^ ^7^, ^9^, ^11^ ^]^ Considering the mechanical properties and angiogenic potential in vitro, we chose D1H1 as an appropriate carrier for VEGF in vivo. We induced hindlimb ischemia in mouse and implanted a dECM/heparin cryogel (D1H1) and VEGF‐loaded dECM/heparin (D1H1_VEGF) to observe blood perfusion recovery. Murine hindlimb ischemia model is commonly used as a model of PAD to test new therapies with new materials or drugs.^[^ ^30^ ^]^ Without any treatment, hindlimb ischemia worsened, and the limb got amputated; however, when treated with dECM/heparin cryogel (D1H1) and VEGF‐loaded dECM/heparin cryogel (D1H1_VEGF), blood perfusion was restored as time passed. As expected, D1H1_VEGF showed the highest blood flow recovery rate, and D1H1 followed. Furthermore, immunohistochemically stained images were in agreement with in vivo results. This is promising because the biomaterial of a scaffold itself provided a neovascularization effect to a certain extent. UBM is widely used as an acellular scaffold and has been known to facilitate tissue regeneration and repair.^[^ ^31^ ^]^ However, all those growth factors and proteins that are preset in UBM may not all be retrieved when going through the decellularization process. There are several ways to decellularize tissue: physical, chemical, and enzymatic protocols.^[^ ^32^ ^]^ Physical decellularization methods such as hydrostatic pressure and sonication damage the ECM structure and do not completely remove DNA fragments.^[^ ^33^, ^34^ ^]^ Chemical and enzymatic approaches using sodium dodecyl sulfate (SDS), Triton X‐100, or trypsin EDTA eliminate the DNA components better than a physical approach, but there remains a possibility of denaturing proteins.^[^ ^34^ ^]^ Because our material was decellularized ECM with a supercritical CO_2_ decellularization technology, we expected that the biomaterial itself would help repair the blood flow. Further detailed studies regarding the composition of supercritical fluid‐based dECM, especially composition of its angiogenic potential, would pave the way for understanding the functional properties of this biomaterial. As this cryogel is easily fabricated and displays a sustained release of VEGF, this has the potential to be used not only for neovascularization but also in various tissue engineering fields with different growth factors.

## 4 Conclusion

In this study, we fabricated a porcine decellularized UBM ECM‐based cryogel as a carrier for VEGF. Varying the concentration of dECM and heparin, we optimized the cryogel suitable for neovascularization. The swelling ratio, interconnected porosity, and Young's modulus were influenced by the concentrations of dECM and heparin as the swelling ratio and interconnected porosity decreased and Young's modulus increased with increasing concentrations of dECM and heparin. All the cryogels were biocompatible and did not adversely affect cell proliferation; however, the cryogels with higher dECM and heparin concentrations showed a slower cell proliferation rate than control and cryogels with lower dECM and heparin concentrations. Between D1H1 and D1H2, lower heparin concentration (D1H1) resulted in more release of VEGF due to their larger pore size and smaller number of heparin‐VEGF binding domain. As we tested angiogenic potential in vitro with the conditioned medium that each cryogel released VEGF for one day, D1H1 group showed a better angiogenic potential than D1H2. In addition to cell scratch assay and tube formation assay to assess angiogenesis, pro‐angiogenic gene expressions of HUVECs increased significantly. In vivo results indicated that VEGF‐loaded dECM/heparin (D1H1_VEGF) exhibited a faster rate of blood vessel regeneration than dECM/heparin cryogel without VEGF (D1H1). Because D1H1 cryogel itself also resulted in some recovery, we could suggest that our cryogel was effective in delivering VEGF for neovascularization. By changing VEGF with other growth factors, our cryogel would be promising for delivering various growth factors for further biomedical applications.

## 5 Experimental Section

### Fabrication of dECM/heparin Cryogels

Supercritical fluid‐based decellularized urinary bladder ECM was kindly provided by DOF Inc, Korea. dECM solution was prepared by dissolving 2% (w/v) dECM in 10% (w/v) pepsin from porcine gastric mucosa (P7125; Sigma–Aldrich, USA) and 0.05 m hydrochloric acid (7647‐01‐0; Duksan, Korea) in deionized water. The solution was stirred for 48 h at 600 RPM. The final solution was neutralized to pH7.4 by adding NaOH, and 1% (v/v) antibiotic‐antimycotic (15 240 062; Thermofisher Scientific, USA). dECM was prepared in concentrations of 1% and 2% (w/v), and heparin (Merck Millipore, H5515) was prepared in 1:1 and 2:1 w/v percentage ratios to dECM solution. 1:10 mass ratio of 4‐(4,6‐Dimethoxy‐1,3,5‐triazin‐2‐yl)‐4‐methylmorpholinium Chloride (DMTMM) (TCI, D2919) to dECM was added to the dECM/heparin solution as a cross‐linker. 70 µL of the mixed solution was added to the cylindrical mold, which is 6 mm in diameter and 2.5 mm in height. The solution was then incubated at −20 °C for 24 h for cryogelation. After the ice crystal formation and cross‐linking reaction were completed, the scaffolds were lyophilized and stored at −20 °C. The final scaffold had a size of 4.5 mm in diameter and 2.5 mm in height.

### Characterization of dECM‐heparin Cryogels

*FT‐IR*: The chemical cross‐linking of dECM/heparin cryogel was analyzed by Fourier‐transform infrared spectroscopy (TENSOR27; Bruker, Germany). Transmittance of dECM, heparin, and dECM/heparin cryogel were measured by scanning 32 times with the resolution of 4 cm^−1^ and the wave number range of 4000–400 cm^−1^. Raw data were baseline‐corrected and normalized.

*Mini‐Scanning Electron Microscopy (Mini‐SEM)*: For preprocessing of mini‐SEM imaging, lyophilized cryogels were cut in half, and the cross‐sections facing up were placed on a stub with a carbon tape. The cross‐sections of cryogels were coated with platinum spluttering at 20 mA for 60 s. The images were taken at 15 kV with 100X magnification with a microscope (JCM‐6000; JEOL, Japan). At least three images per sample were taken. The diameter of the pores was measured by ImageJ software (https://imagej.nih.gov/ij/).

*Swelling Ratio*: Each of the fully lyophilized dECM‐heparin cryogel was weighed and swelled in 500 µL of distilled water. After 24 h incubation at 37 °C, the swollen cryogel was weighed on a scale. The swelling ratio was calculated as follows.

(1) $$Swelling\;Ratio=\frac{Swollen\;weight-Initial\;weight}{Initial\;weight}$$

*Interconnected Porosity*: Masses of the cryogel were first measured in their swollen state. After dehydration, the final masses were measured. The interconnected porosity was calculated as follows:

(2) $$\begin{array}{ccc} & & Interconnected\;Porosity\\ & & \;=\frac{Dehydrated\;cryogel\;weight-Swollen\;cryogel\;weight}{Swollen\;cryogel\;weight}\times 100\% \end{array}$$

*Compression Test*: Young's modulus was calculated based on the compression test result measured by Universal Testing Machine (UTM; Shimadzu EZ‐SX, Japan). The diameter and height of each cryogel were measured using a digital Vernier caliper. The cryogels were compressed with the loading speed of 5 mm min^−1^. The stress–strain curve was plotted, and Young's modulus was calculated from the linear region of the graph.

*Collagenase Degradation Assay*: Degradation rate of cryogels were calculated based on the percentage of remaining weight of cryogels after incubating in 0.2 unit mL^−1^ or 1 unit mL^−1^ of collagenase type II (Worthington, USA) in PBS at 37 °C for 4 days. The degradation rate was calculated as follows.

(3) $$Degradation\;rate=\frac{Remaining\;cryogel\;weight}{Original\;cryogel\;weight}\times 100\%$$

### Rheology Test

Two rheology tests had been conducted with Advanced Rheometric Expansion System (ARES; Rheometric Scientific, UK): strain sweep and frequency sweep. The strain sweep test was conducted to measure the stability of cryogel against deformation. The strain range of 1 – 100% at the constant frequency of 6.3 rad s^−1^ was used. The frequency sweep test was conducted to measure the cross‐linking degree with a stiffness property. 0.1 to 100 rad s^−1^ at a constant strain of 5% were set as a parameter. The cryogel used throughout the rheology test was 8 mm in diameter and 2 mm in height. Prior to rheological studies, cryogels were allowed to swell in PBS.

### Biocompatibility Assay

Cytotoxicity of dECM/heparin cryogels and cell proliferation with cryogels were tested. For these assays, Human Umbilical Vein Endothelial Cells (HUVECs) in their passages 4–7 and the conditioned medium were used. To collect the conditioned medium, each dECM/heparin cryogel was sterilized with 3% Antibiotic‐Antimycotic (15 240 062; Gibco, USA) for 1 h and washed with Dulbecco's Phosphate Buffered Saline (DPBS) w/o Calcium w/o Magnesium (L0615; Biowest, USA) for 1 h. 1 mL of Endothelial Cell Growth Medium MV2 (EGM2) (C‐22022; Promocell, Germany) was added to each of the sterilized cryogel. The medium was collected after 24 h and filtered with 0.2 µm syringe filter (SP13P020SL, Hyundai Micro). For LIVE/DEAD assay, 10 000 cells cm^−2^ were seeded in a 96‐well plate. After the cells were attached to the well plate, the conditioned medium was replaced from EGM2. After 24 h, LIVE/DEAD solution composed of 0.5 µL mL^−1^ Calcein‐AM (C3099; Thermofisher Scientific, USA) and 2 µL mL^−1^ Ethidium Homodimer‐1 (E1169; Thermofisher Scientific, USA) was added to well plate and incubated for 15 minutes in a humidified incubator at 37 °C with 5% CO_2_. Cells were imaged with EVOS2 (Thermo Fisher Scientific, USA). For cell proliferation assay, cell counting kit‐8 (CCK8) (CK04‐13; Dojindo, Japan) was used. 10000 HUVECs cm^−2^ were seeded in a 96 well plate and were provided with a conditioned medium after cells were attached to the well plate. After 24 h of incubating with a conditioned medium, 10% CCK8 solution was added to each well and incubated for 2 h in a humidified incubator at 37 °C with 5% CO_2_. The absorbance at 450 nm was measured with a microplate reader (TECAN, Switzerland). After the measurement, the cells were washed with PBS and provided with a fresh conditioned medium. The cell proliferation was measured every day for 3 days.

### Cell Scratch Assay

Cell scratch assay was conducted to show cell migration upon an addition of human VEGF165 (RVEGFI, Invitrogen, USA). The conditioned medium was made as follows. 500 ng of VEGF‐containing cryogels were incubated in the basal medium (EBM) (C22022, Promocell, Germany) supplemented with 2% Fetal bovine serum (FBS) (35‐015‐CV; Corning, USA) and 1% antibiotic‐antimycotic at 37 °C incubator for 24 h. The conditioned medium was collected and filtered with a syringe filter (SP13P020SL; Hyundai Micro, Korea). HUVECs were seeded on a 24 well plate, cultured until confluence and thereafter starved with EBM for 2 h. After scratching the surface of the plate with a 1000 µL pipette tip, cells were washed with DPBS once and provided with the conditioned medium. Cells were imaged with EVOS2 after 12 h.

### Tube Formation Assay

Tube formation assay was done to examine the ability of HUVECs to form tubular network upon an addition of VEGF. Growth factor reduced Matrigel (354 230; Corning, USA) was thawed on ice at 4 °C overnight before the use. 35 µL of Matrigel was coated on each well of a precooled 96 well plate and incubated for 30 min at 37 °C incubator. HUVECs were starved with EBM for 6 h and trypsinized (25200‐114; Gibco, Germany) for the study. 15 000 cells were seeded in each well with 100 µL of the conditioned medium. The conditioned medium was the same as the medium used for the cell scratch assay. After 12 h of incubation, cells were stained with 0.5 µL mL^−1^ of Calcein‐AM. Tube formation was imaged in both bright field and fluorescence with EVOS2. The numbers of meshes, nodes, branches, and junctions were measured via ImageJ.

### VEGF Releasing Rate

To evaluate the VEGF‐release profile of the cryogels, 10 µL of PBS containing VEGF solution (100 ng mL^−1^) was added to each cryogel. VEGF were immobilized to the cryogels at −20 °C for 24 h and subsequently lyophilized overnight. The cryogels were then rinsed with PBS to remove unbound VEGF. Following this, 500 µL of DPBS was added to each VEGF‐loaded cryogel, and aliquots were collected at pre‐determined time points over a 14‐day period. The concentration of released VEGF was quantified using the Human VEGF ELISA Kit, following the manufacturer's protocol (K0331132; LABIS KOMA, Korea).

### Reverse Transcription Quantitative Real‐time Polymerase Chain Reaction (RT‐qPCR)

RT‐qPCR was conducted to compare the angiogenic gene expressions on HUVECs in response to dECM/heparin cryogel loaded with VEGF. HUVECs seeded on a 12‐well plate were co‐cultured with the dECM/heparin cryogel loaded with VEGF for three days. RNAiso (9109; Takara, Japan) was then added to cells for RNA extraction. Chloroform (C2432; Sigma–Aldrich, USA) separated the RNA from DNA and debris, and RNA was recovered as a precipitate from the solution with isopropanol (I9516; Sigma–Aldrich, USA). After denaturing RNA in a 58 °C‐heat block for 15 min, dNTP, reverse transcriptase, RNase inhibitor, random hexamer, and buffer (Enzynomics, Korea) were mixed with samples to synthesize cDNA using MiniAmp Thermal Cycler (Thermofisher Scientific, USA). For qPCR, SYBR green (Enzynomics, Korea) was used as a dye, and it was run with StepOnePlus (Applied Biosystems, USA).

### In Vivo Hindlimb Ischemia

Animal experiments were approved by the Institutional Animal Care and Use Committee at Seoul National University (IACUC; SNU‐230904‐2‐1). The hindlimb ischemia model was induced following the protocol from previous studies.^[^ ^7^, ^11^, ^15^ ^]^ Briefly summarizing, an eight‐week‐old BALB/c‐nu mouse (Narabiotech, Korea) was anesthetized by inhalation of isoflurane (3010; Hana Pharm, Korea), and a small incision was made on the inguinal skin of the hind limb. The femoral artery was ligated with the silk suture (SK324; Duksan, Korea) at the proximal and distal sites, and it was completely excised with a cautery pen (Bovie Medical Corporation, USA). Mice were then treated with either PBS, a dECM/heparin cryogel, or VEGF‐loaded dECM/heparin cryogel. After suturing the skin, the blood perfusion was analyzed with Laser Doppler Imaging System (moorLDI2; Moor Instruments, UK) on day of surgery, day 3, 7, 14, 21, and 28. The LDPI index was plotted to express blood perfusion level, and the salvage level was scored where (0: full recovery; 1: toe discoloration; 2: toe discoloration; 3: foot discoloration;, 4: toe amputation; 5: foot amputation; 6: limb amputation).

### Paraffin Sectioning and Immunohistochemistry

Mice were sacrificed on day 28 with CO_2_, and the hindlimb tissue were collected and fixed with 4% paraformaldehyde (PFA) (6506‐4405; Daejung Chemicals & Metals, Korea) at 4 °C for 4 days. The tissue was then moved to cassettes and were washed with tap water overnight to remove PFA. Dehydration steps were done by dipping the tissue in deionized water, 50% EtOH, 70% EtOH, 90% EtOH, 100% EtOH twice, and xylene twice for 30 min in each step. The tissue was then incubated in a paraffin solution overnight at 60 °C. The tissue was embedded in paraffin blocks and sectioned in 10 µm thickness with a microtome (Leica, USA). After rehydration steps with the aforementioned xylene and decreasing ethanol concentration solutions for 5 min each, sectioned tissue was stained immunohistochemically. Heat‐induced epitope retrieval was done with citric buffer (C9999; Sigma–Aldrich, USA) at 100 °C for 10 min with an autoclave. Permeabilized and blocked sectioned tissue was incubated with the primary anti‐mouse alpha smooth muscle actin antibody (ab5694; Abcam, UK) diluted to 200:1 overnight at 4 °C. Then, secondary goat anti‐rabbit IgG (AB150077; Abcam, UK) diluted to 500:1 was applied for 1 h at room temperature. Samples were mounted with fluoroshield mounting medium with DAPI (ab104139; Abcam, UK) and imaged under the fluorescence microscope (Nikon TE2000‐U Japan). Three mice per group were used, with one slide per mouse and three images per slide. α‐SMA‐stained blood vessels were counted, summed, and averaged across three replicates.

### Hematoxylin‐Eosin Staining

After the dehydration step, which is the same as 2.10., the sectioned tissue was stained with hematoxylin (H3404‐100; Vector Laboratories, USA) for 3 min and rinsed with tab water for 5 min. Eosin staining was done with 0.25% eosin (318 906; Sigma–Aldrich, USA) in acetic acid (K33417963; Sigma–Aldrich, USA) for 45 s. The sectioned tissue was then rehydrated by dipping the slide glasses five times in tab water, 70% EtOH, 90% EtOH, 100% EtOH twice, and xylene twice. A drop or two of DPX mountant (06522; Sigma–Aldrich, USA)was added on a stained tissue, and it was covered with coverslip slides. The images were taken under the bright field microscope (Nikon TE2000‐U Japan).

### Statistical Analysis

Statistical analysis was performed using GraphPad Prism version 8. One‐way and two‐way ANOVA were used for statistical analyses.

## Conflict of Interest

The authors declare no conflict of interest.

## Supporting information



Supporting Information

## Data Availability

The data that support the findings of this study are available from the corresponding author upon reasonable request.
