# Supplementary material for: Development of a Decellularized Urinary Bladder Matrix and Heparin‐Based Cryogel for Promoting Angiogenesis
Source: Macromol Biosci. 2025 Apr 30;25(8):2500028. doi: 10.1002/mabi.202500028 (PMC12351666; doi:10.1002/mabi.202500028)
Supplement: Supplementary file 1 — Supporting Information [file MABI-25-2500028-s001.docx]

**Supporting Information**


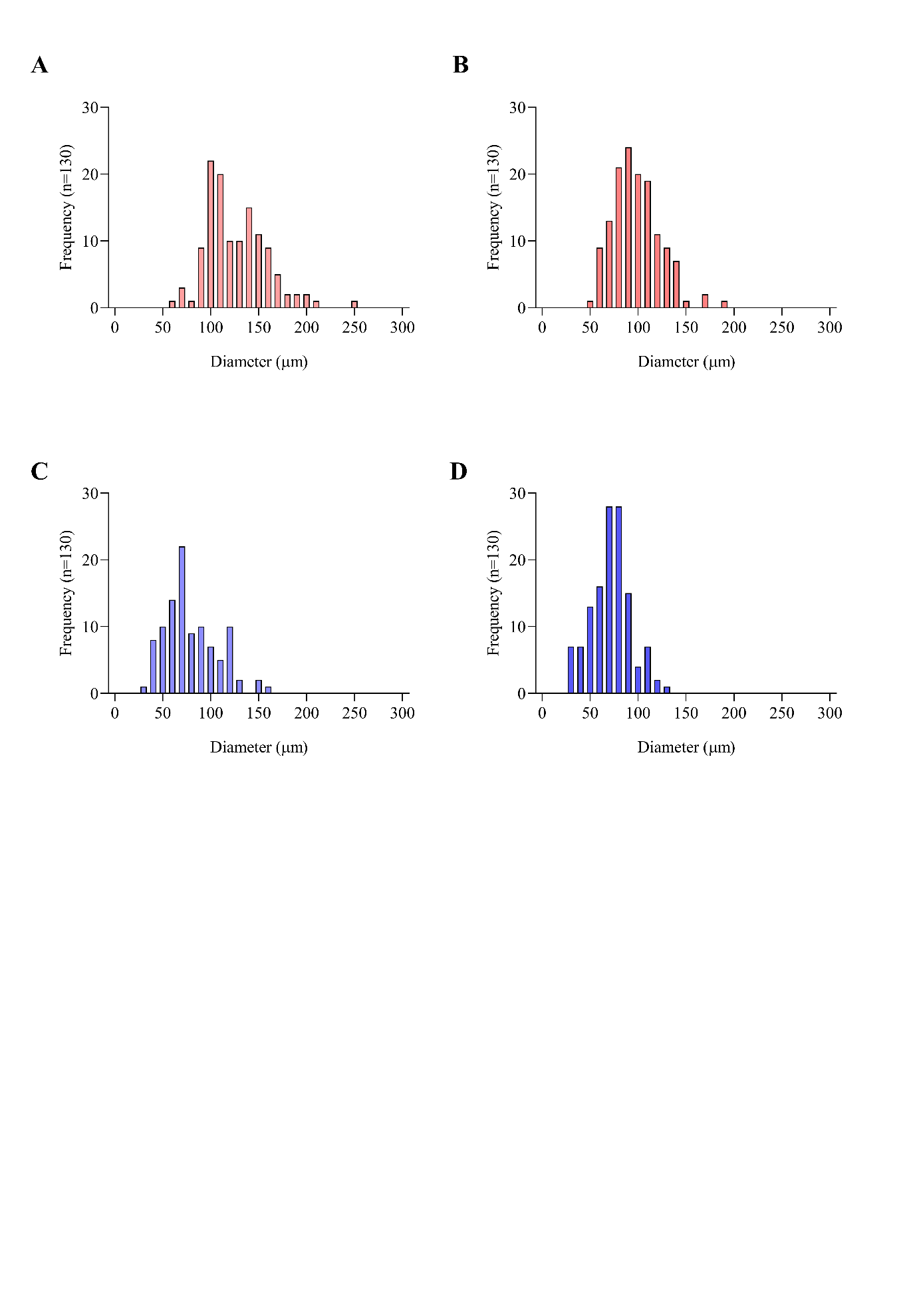


Figure S 1. Frequency distribution of pore diameter.

Pore distribution of (A) D1H1 cryogel, (B) D1H2 cryogel, (C) D2H2 cryogel, and (F) D2H4 cryogel.


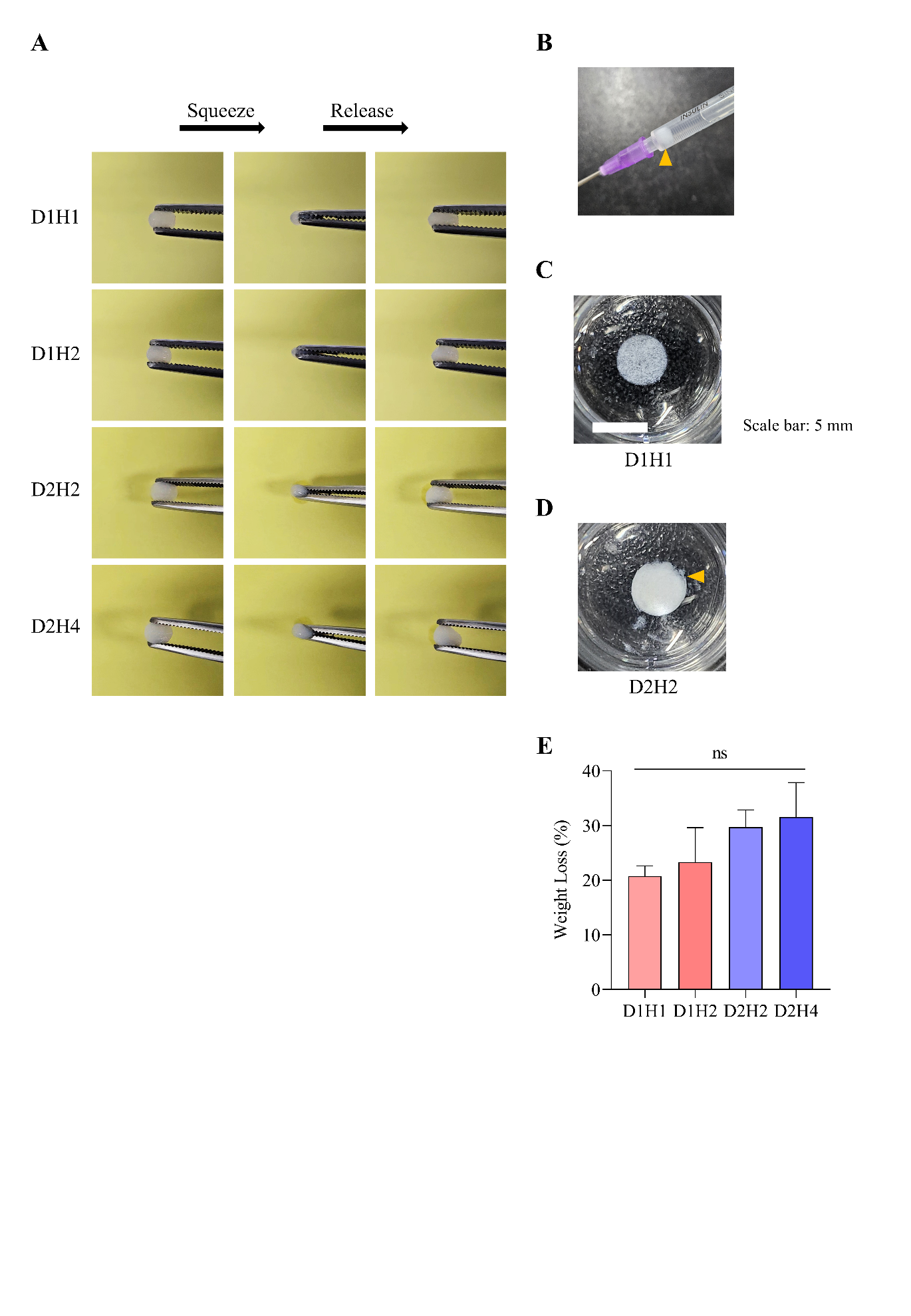


Figure S 2. Injection potential of dECM/heparin cryogels.

(A) Images of cryogels recovering their shapes after squeezing. (B) An image depicting the injection of dECM/heparin cryogel with a 1 mL syringe and 17G needle. Images of (C) D1H1 cryogel and (D) D2H2 cryogel after injection, and (E) the quantification of weight loss after injection. The scale bar was 5 mm.


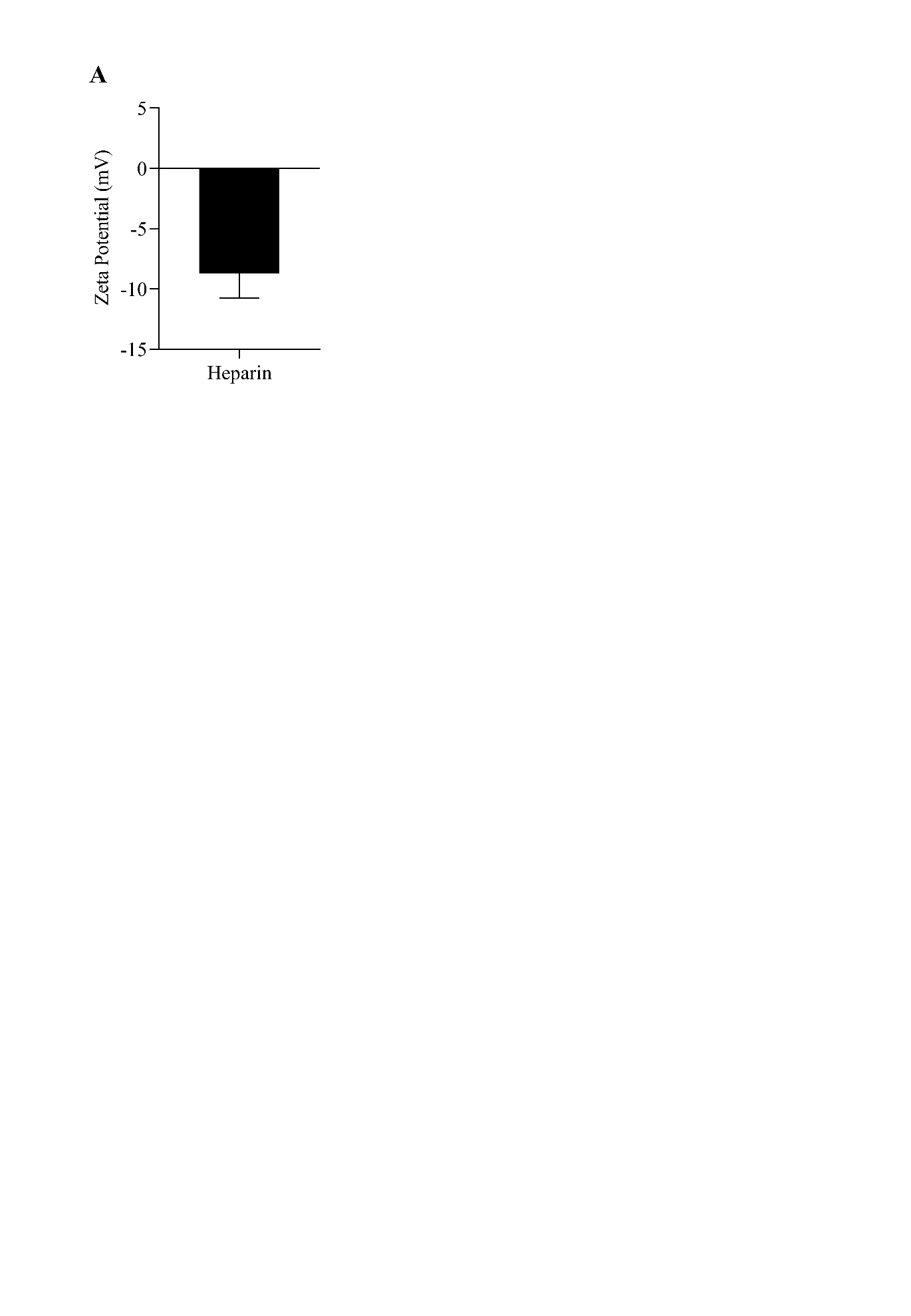


**Figure S 3. Zeta potential of heparin.**

**
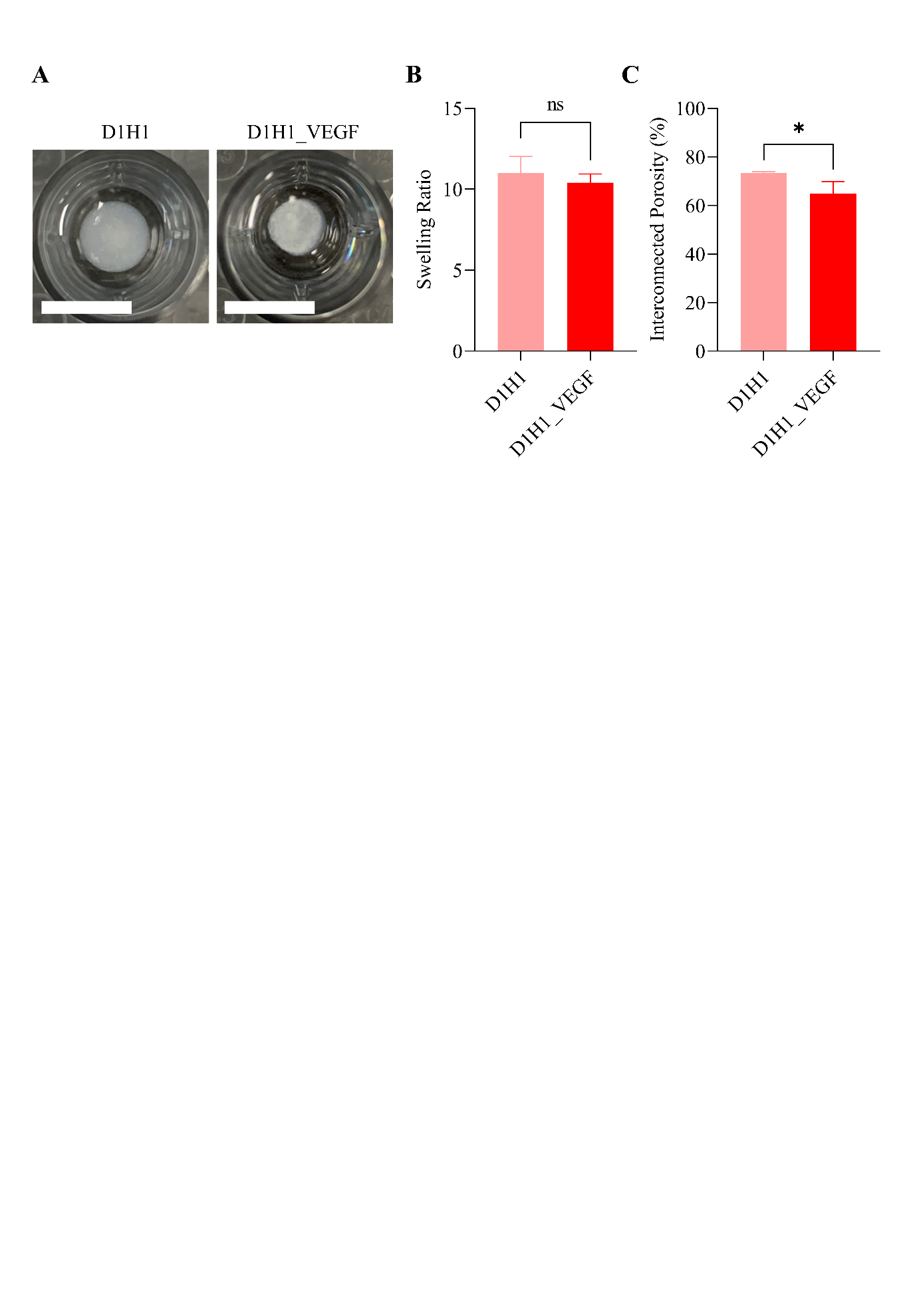
**

**Figure S 4. Characterization of VEGF-unloaded and VEGF-loaded dECM/heparin cryogels.** (A) Macroscopic images of VEGF-unloaded dECM/heparin cryogel (D1H1) and VEGF-loaded dECM/heparin cryogel (D1H1_VEGF). (B) Swelling ratio and (C) interconnected porosity of D1H1 and D1H1_VEGF. The scale bar was 5 mm.
